# Nutrition protocols improve caloric adequacy in critically Ill children: a systematic review and meta-analysis

**DOI:** 10.3389/fped.2026.1889905

**Published:** 2026-07-14

**Authors:** Yan Wang, Jia Wang, Xiao Sun, Baolian Yao, Fang Guo, Yan Shi, Nida Naeem

**Affiliations:** 1 Department of Critical Care Medicine, Children's Hospital of Xinjiang Uygur Autonomous Region, Xinjiang Hospital of Beijing Children's Hospital, The Seventh People's Hospital of Xinjiang Uygur Autonomous Region, Pediatric Research Institute of Xinjiang Uygur Autonomous Region, Urumqi, China; 2 Institute of Pharmaceutical Sciences, University of Veterinary and Animals Sciences, Lahore, Pakistan

**Keywords:** caloric adequacy, critical illness, enteral nutrition, nurse-led protocol, nutritional support, pediatric intensive care

## Abstract

**Background:**

Malnutrition is common among critically ill children admitted to the Pediatric Intensive Care Unit (PICU). Despite established guidelines, real-world caloric delivery consistently falls short. Structured nutritional protocols have been proposed to close this gap, yet no prior meta-analysis has quantified their pooled effect on caloric adequacy specifically in PICU patients.

**Methods:**

We searched through PubMed, Embase, the Cochrane Library, and Web of Science from January 2015 to March 2026. Studies enrolling critically ill children (≤18 years) in the PICU with a comparison group and at least one nutritional delivery outcome were eligible. The primary outcome was caloric adequacy. A random-effects model was used for pooling effect. Subgroup analyses, meta-regression, sensitivity analyses, and publication bias assessments were conducted.

**Results:**

Nine studies enrolling 1,992 children across six countries were included. Nutritional protocols were associated with a pooled mean difference of +21.98% in caloric adequacy (*p* < 0.001), with a substantial heterogeneity (I^2^ = 73.9%). Both nurse-led (MD +19.16%) and non-nurse-led protocols (MD +23.95%) improved delivery without a significant between-group difference (*p* = 0.51). Study design was the dominant heterogeneity driver and explained 93.6% of between-study variance. The pooled estimate was robust across all sensitivity analyses, and trim-and-fill imputed zero missing studies.

**Conclusions:**

Structured nutritional protocols meaningfully improve caloric adequacy in critically ill children, regardless of whether nurses lead their implementation. These findings support the wider adoption of protocolized feeding as a standard of care in PICUs globally.

## Introduction

Critically ill children admitted to the Pediatric Intensive Care Unit (PICU) represent a highly vulnerable population with increased metabolic demands and a high risk of malnutrition (1). This susceptibility is exacerbated by the catabolic state induced by critical illness, leading to rapid depletion of endogenous energy stores and lean body mass (2). The prevalence of malnutrition upon PICU admission is notably high and is independently associated with adverse outcomes, including prolonged mechanical ventilation, increased susceptibility to nosocomial infections, and elevated mortality rates (3).

Caloric deficits accumulate rapidly during PICU admission due to the hypermetabolic catabolic state, characterized by increased resting energy expenditure (4). Cumulative negative energy balance from such underfeeding is associated with prolonged mechanical ventilation and fewer ventilator-free days (5), heightened risk of hospital-acquired infections (6), and increased 60-day mortality (7). Even short-term caloric deficits within the first week of PICU admission, have been shown to result in significant muscle mass loss (8). Furthermore, failure to achieve target calorie intake within the initial 48 h and at seven days of PICU admission correlates with a higher mortality rate (9). However, the optimal timing and modality of nutritional provision, especially concerning the balance between enteral and parenteral routes and the impact of feeding protocols, remain subjects of ongoing debate.

Enteral nutrition is physiologically beneficial. Maintenance of gut integrity and reduced infectious complications (10) are favored by enteral nutrition. However, its timely and adequate delivery is frequently hindered by numerous barriers (11). European Society of Pediatric and Neonatal Intensive Care (ESPNIC) (4) guidelines recommend early enteral nutrition initiation within 24–48 h and stepwise advancement to 60%–100% of energy targets (e.g., Schofield equation-based 25–30 kcal/kg/day). Yet, the real-world PICU delivery averages 34 kcal/kg/day or 45%–66% of goals, with 38% of patients remaining calorically deficient (3). This gap stems from four primary barriers: prolonged procedural fasting, perceived feeding intolerance, absence of standardized protocols and clinician-level variability. These systemic and individual factors collectively contribute to inadequate caloric provision, thereby highlighting an urgent need for standardized, evidence-based nutritional protocols to optimize care delivery in this vulnerable population.

To address these multifaceted barriers to adequate caloric provision, structured nutritional feeding protocols have been developed as targeted interventions in PICUs (4). These protocols span a spectrum of designs, including nurse-led approaches that empower bedside staff to initiate and titrate feeds based on predefined criteria (12, 13), pharmacist-involved nutrition support teams coordinating parenteral supplementation and monitoring (14, 15), and algorithm-driven strategies that standardize progression toward caloric goals while minimizing interruptions (4, 16). While individual studies and narrative reviews have demonstrated their feasibility and benefits in enhancing nutrient delivery and time to goal feeds (13, 16, 17), no prior systematic review or meta-analysis has quantified the pooled effect on caloric adequacy specifically in critically ill pediatric patients within the PICU. We hypothesized that structured nutritional protocols would significantly improve caloric adequacy compared with standard nutritional practices in critically ill children admitted to the PICU. Therefore, we pooled data from these individual studies to evaluate the efficacy of nutritional protocols in achieving caloric targets and improving clinical outcomes among critically ill pediatric patients in the PICU.

## Methods

### Literature search and study selection

**Figure 1:**
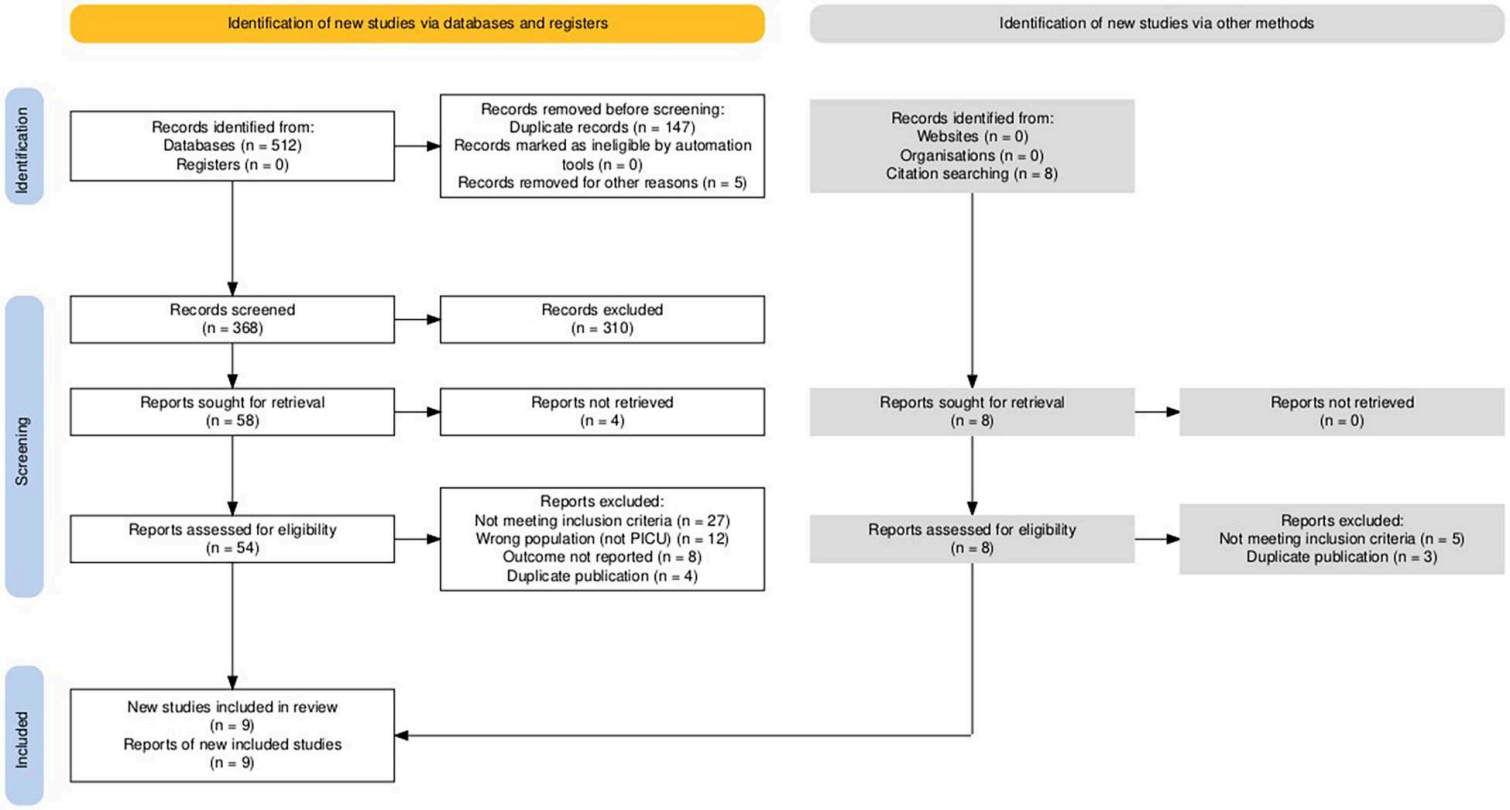
PRISMA flowchart for screening and identifying studies searched from databases.

PRISMA guidelines (18) were followed during literature search and study selection. A systematic search across PubMed, Web of Science, the Cochrane Library, and Embase was conducted from January 2015 to March 2026. The search was restricted to publications in the English language. Complete literature search criteria and keywords used are tabulated in Supplementary Table S1. Titles and abstracts identified through database searches were screened against eligibility criteria. Full texts of potentially eligible records were retrieved and assessed independently. Disagreements at any stage of screening were resolved by consensus between reviewers. Studies were eligible (Figure 1) if they enrolled critically ill children (≤18 years) admitted to a PICU, with at least one nutritional delivery outcome reported. Eligible designs included RCTs, quasi-experimental, pre-post, and cohort studies with a comparison group. Excluded studies included studies from NICUs, general wards, or adult ICUs, and single-arm studies. In mixed PICU/NICU populations, only studies reporting PICU data separately were included. Cardiac PICUs were considered eligible.

### Data extraction

A standardized data extraction form was used to collect the following variables from each eligible study: author, publication year, country, study design, study start and end dates, study duration, ICU type, total sample size, intervention group size, control group size, patient age (median and interquartile range), sex, number of patients receiving mechanical ventilation, ventilation duration, protocol type (nurse-led vs. non-nurse-led), comparator type, enteral feed route, caloric adequacy in the intervention and control group, and the reported *p*-value for the between-group comparison. The primary outcome for quantitative synthesis was caloric adequacy, defined as the percentage of prescribed caloric target delivered during the study duration over which caloric intake was measured in each included study, typically from PICU admission until PICU discharge. Per-study effect sizes were expressed as the mean difference (MD) in caloric adequacy percentage between the groups.

### Quality appraisal

The quality of included studies was assessed using the Newcastle-Ottawa Scale (NOS) for non-randomized observational studies. Studies were evaluated across, selection of study groups, comparability of groups, and ascertainment of outcomes by a score out of nine, with seven or more stars indicating low risk of bias.

### Publication bias and sensitivity analyses

Publication bias was assessed using Egger's test for funnel plot asymmetry and the trim-and-fill method of Duval and Tweedie. Given that the total number of included studies was fewer than ten, statistical power for detecting asymmetry was limited; results of publication bias tests were therefore interpreted with caution.

Five sensitivity analyses were conducted to examine the pooled estimates. A leave-one-out analysis, separate pooling restricted to interventional studies (*k* = 4) only and to observational studies (*k* = 5) only. Re-estimation using DerSimonian-Laird and Paule-Mandel *τ*^2^ estimators were employed to confirm that the results were not dependent on the estimation method. Meanwhile. restriction to studies with a concurrent standard care comparator (*k* = 5) was chosen as sensitivity analysis to determine whether secular trends in nutritional practice could account for the pooled effects.

### Statistical analysis

All statistical analyses were conducted using Python v3.12.13 (March 2026). Medians with interquartile ranges were converted means and standard deviations (according to Wan et al. (19),) before pooling. Per-study standard errors were derived from 95% *p* values in case of unavailability of standard deviations as per Altman and Bland (20). A two-tailed *p* value < 0.05 was used to define statistical significance. A random-effects model with restricted maximum-likelihood (REML) estimation of the between-study variance (*τ*²) was used to calculate pooled MD with its 95% confidence interval. Heterogeneity was quantified Q statistic, the I² statistic, and the between-study standard deviation *τ*. I² values of approximately 25%, 50%, and 75% were taken as indicative of low, moderate, and substantial heterogeneity, respectively. Subgroup analyses were conducted with subgroup strata, including protocol type, comparator type and study design. A univariable random-effects meta-regression was conducted to identify covariates associated with between-study variation in the MD. Covariates examined individually included: protocol type, comparator type, study design, enteral feed route, patient age, study duration, and total sample size. Given the small number of studies (*k* = 9), no more than two covariates were entered simultaneously in any single model. The proportion of heterogeneity explained by each model was quantified using an R² analogue. Knapp-Hartung confidence intervals were used throughout. Bivariable models were constructed for pairs of covariates with low collinearity (|r| < 0.5), including protocol type and age, comparator type and study design, and protocol type and study duration.

## Results

### Characteristics of included studies

A total of 9 studies (*n* = 9, tabulated in Table 1) were included in pooled analysis which comprised of 1,992 pediatric patients. The studies represent reasonably diverse geography as conducted across 6 different countries. Slightly more than half were observational (*n* = 5, 55.6%), while the remaining were interventional studies (*n* = 4, 44.4%). Study durations and nutritional protocols varied between studies, with noticeable heterogeneity in intervention approaches and comparator, which influence downstream pooled interpretations. The reported median age ranges from infants (3 months) to older pediatric patients (88.8 months) and a total of 949 participants were male which suggests a modest male predominance. A substantial proportion of patients (*n* = 836, −42.0% of total sample) undergone mechanical ventilation reflect the clinical severity of the included populations.

**Table 1:** Summary of demographic and clinical characteristics of included studies.

Author	Country	Study design	Study duration (years)	Sample size	Male/female ratio	Median age (IQR)	Patients undergone ventilation	Protocol type	Type of Comparator	Enteral feed route
Geukers et al., (14)	Netherlands	Observational	4.91	171	0.9	4.7 (0.9–18.9)	87	Nurse-led protocol	Historical Control	Gastric
Hamilton et al., (21)	USA	Interventional	3.92	160	1.05	78 (18–180)	36	Non–nurse-led	Standard care	Gastric
Ang et al., (12)	Singapore	Observational	1.42	40	0.48	9.4 (2.8–57)	31	Nurse-led protocol	Standard care	Nasogastric
Ziemba et al., (22)	USA	Interventional	4.08	648	0.62	3 (1–9)	272	Non–nurse-led	Historical Control	Nasogastric
Sng et al., (23)	Singapore	Interventional	5.24	167	0.44	14 (4–49)	92	Nurse-led protocol	Standard care	Nasogastric
Zeeshan et al., (24)	Pakistan	Interventional	1.91	180	1.57	48 (12–132)	81	Non–nurse-led	Standard care	Nasogastric
Rungsattatharm et al., (25)	Thailand	Observational	1.67	215	1.34	16 (6–69)	155	Non–nurse-led	Historical Control	Nasogastric
Ceci et al., (26)	France	Observational	7.92	141	1.47	88.8 (43.2–135.6)	58	Non–nurse-led	Historical Control	Nasogastric
Huq et al., (27)	USA	Observational	2.33	270	1.41	3 (2–7)	24	Non–nurse-led	Standard care	Nasogastric

### Intervention improves caloric adequacy

**Figure 2:**
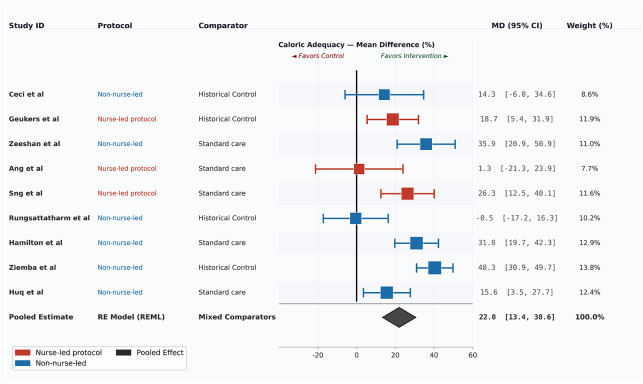
Effect of interventions on caloric adequacy across included studies. Forest plot of mean difference in caloric adequacy (%), with study weights and 95% CIs shown; values favor intervention to the right, and the pooled random-effects estimate indicates improved adequacy (MD 22.0%, 95% CI 13.4–30.6).

The delivery of caloric intake was consistently higher in the intervention groups compared with controls. In pooled analysis (Figure 2), the intervention was associated with an absolute increase of 21.98% in caloric adequacy (*p* < 0.001). In practical, most studies showed a clinically meaningful improvement in achieving prescribed nutritional targets, reporting gains exceeding 30%, particularly in nurse-led protocol settings. However, this effect was not uniform with studies (*n* = 2) showing minimal difference and a study even showing a slight negative effect which would suggest that local practices, feeding protocols, and patient acuity likely influenced outcomes. This prediction interval (−0.89% to 44.86%) indicates that while overall benefit is likely in most situations, it can be modest or even non-existent, in some clinical situations. But the big picture clearly skews toward better caloric delivery with intervention, and in mechanically ventilated patients where nutritional deficits are common. Overall heterogeneity (*I^2^* = 73.9%) shows that implementation factors, rather than the intervention itself, probably drive much of the difference observed across the studies.

### Subgroup analyses

**Figure 3:**
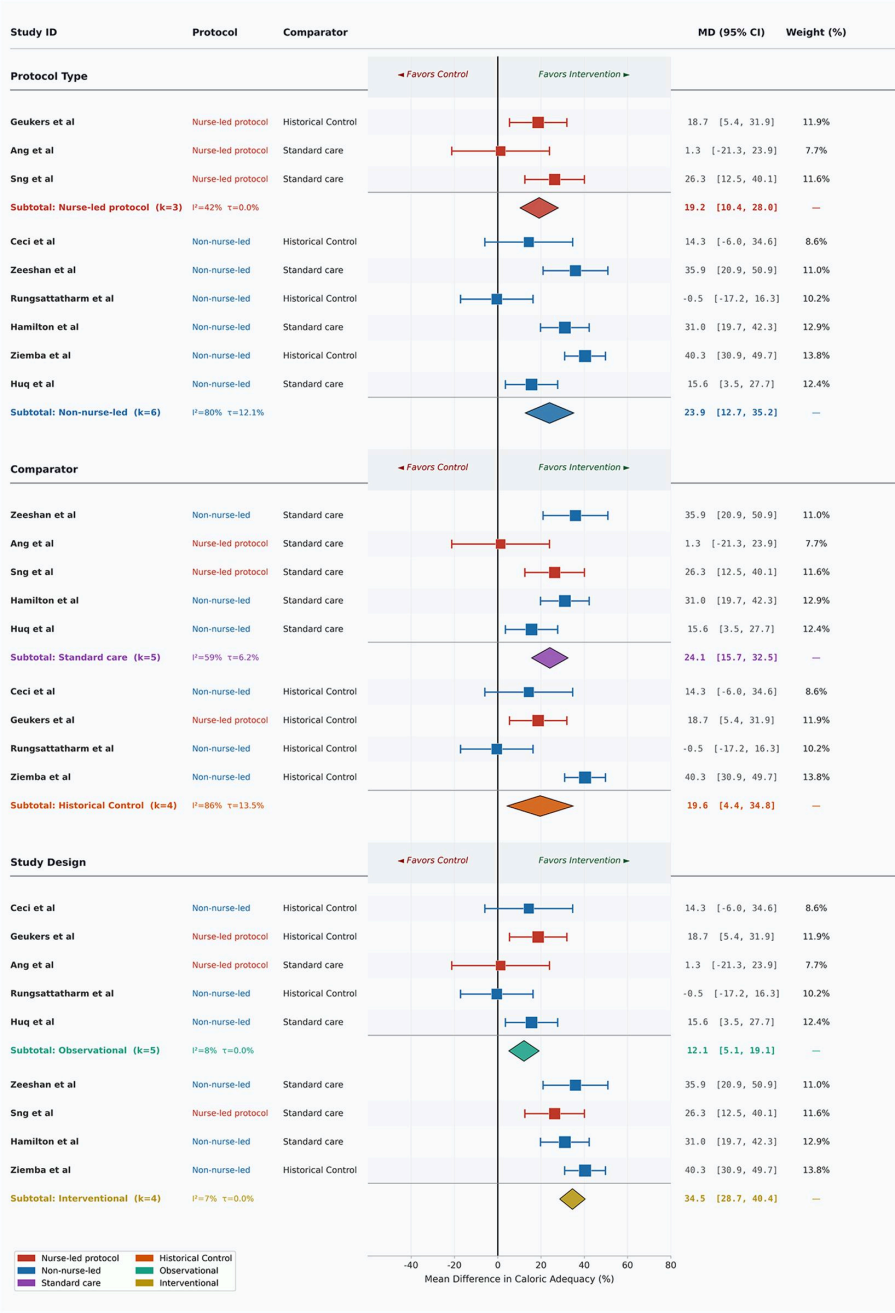
Subgroup analysis of interventions on caloric adequacy. Forest plot showing subgroup analyses by protocol type, comparator, and study design, with pooled estimates indicating consistently higher caloric adequacy with intervention, particularly in interventional studies.

Stratification by protocol type revealed that benefit is not strictly dependent on who leads the protocol (Figure 3), but rather on the presence of a structured nutritional strategy itself as both nurse-led [*k* = 3, MD = +19.16% (95% CI 10.36 to 27.95)] and non–nurse-led approaches [*k* = 6, MD = +23.95% (95% CI 12.71 to 35.19)] were associated with improved caloric adequacy. No statistically significant difference between these groups was observed (*p* = 0.51). Similarly, interventions outperformed both standard care (k = 5, MD =  +24.09%, 95% CI 15.68 to 32.49) and historical controls (*k* = 4, MD = +19.60%, 95% CI 4.36 to 34.84), however, without a meaningful difference (*p* = 0.61). The direction of effect remained consistent, though variability was more pronounced in studies using historical controls which points at less stable baseline practices in earlier cohorts.

In contrast, study design appeared to influence the magnitude of effect. Interventional studies demonstrated a substantially larger improvement in caloric adequacy (*k* = 4, MD = +34.54%, 95% CI 28.66 to 40.42) compared with observational studies (*k* = 5, MD = +12.14%, 95% CI 5.15 to 19.13), with a clear difference between groups (*p* < 0.001). This divergence is likely because of more strict protocol adherence and more controlled implementation in real interventional settings as opposed to the most practical constraints in the real-world setting in which this observational data was collected.

### Meta-regression

Meta-regression (Table 2) was conducted cautiously due to limited number of studies (*k* = 9) and restricted the models ≤2 covariates at a time. Univariable analyses indicated that the predefined covariates including protocol type, comparator, age, enteral feeding route, study duration, and total sample size, at least in isolation, were not major drivers of between-study variability. This is because, these covariates did not show a statistically meaningful association with the pooled effect estimate (all *p* > 0.10) and heterogeneity for these models remained negligible (R^2^ ≈ 0%). In contrast, study design emerged as the only covariate with a clear signal. Interventional studies were associated with a significantly larger effect size compared to observational designs (*β* =  + 22.39, SE = 4.89; *p* = 0.0025) which explains a substantial proportion of heterogeneity (R^2^ = 93.6%) and significantly reducing residual variance (*τ*^2^ = 7.54). This pattern persisted in further bivariable analysis when adjusted for comparator type (*β* = +23.41, SE = 4.91; *p* = 0.0031; R^2^ = 97.5%). These findings reinforce the robustness of these models.

**Table 2:** Meta-regression analyses exploring sources of heterogeneity.

Covariate	*β*	SE	*p*-value	95% CI	R² (%)	*τ*²
Univariable models						
Nurse-led protocol	−6.94	10.5	0.5298	−31.78 to 17.89	0	154.57
Standard care comparator	3.48	10.05	0.7393	−20.28 to 27.23	0	165.98
Interventional study design	22.39	4.89	0.0025	10.83 to 33.94	93.6	7.54
Gastric route of enteral feed	4.47	11.63	0.7124	−23.03 to 31.97	0	167.48
Age (month)	0.07	0.16	0.6838	−0.31 to 0.44	0	162.8
Study duration (years)	1.56	2.62	0.5714	−4.64 to 7.75	0	153.89
Sample size	11.53	6.33	0.1111	−3.43 to 26.49	18.1	95.76
Bivariable models						
Protocol *vs* Age	−6.12/ +0.03	NA	>0.60	ns	0	190.43
Comparator *vs* Design	−5.20/ +23.41	NA	0.326/0.0031	NA	97.5	2.91
Protocol *vs* Duration	−8.02/ +1.84	NA	>0.49	ns	0	165.63

Clinically, this divergence is likely to reflect differences in structure and not true biological modification of effect, for example, interventional studies may include more uniform protocols, better adherence and possibly select inclusion of patients, which can all inflate observed efficacy. Meanwhile, non-association with median age (*β* = +0.07 per month; *p* = 0.68) is somewhat interesting given the broad age range (3–88.8 months) which suggests the response to treatment was relatively constant across different pediatric subgroups.

Similarly, protocol type did not show a measurable effect (*β* = −6.94; *p* = 0.53) from a clinical workflow perspective, which is a little surprising. But with a small sample and heterogeneity, it is hard to see subtle effects of implementation. Lastly, total sample size showed a non-significant trend (*β* = +11.53; *p* = 0.11; R^2^ = 18.1%). This shows that smaller studies might be contributing to effect size dispersion. Therefore, other than study design, no single covariate showed a plausible explanation for heterogeneity.

### Publication bias

**Figure 4:**
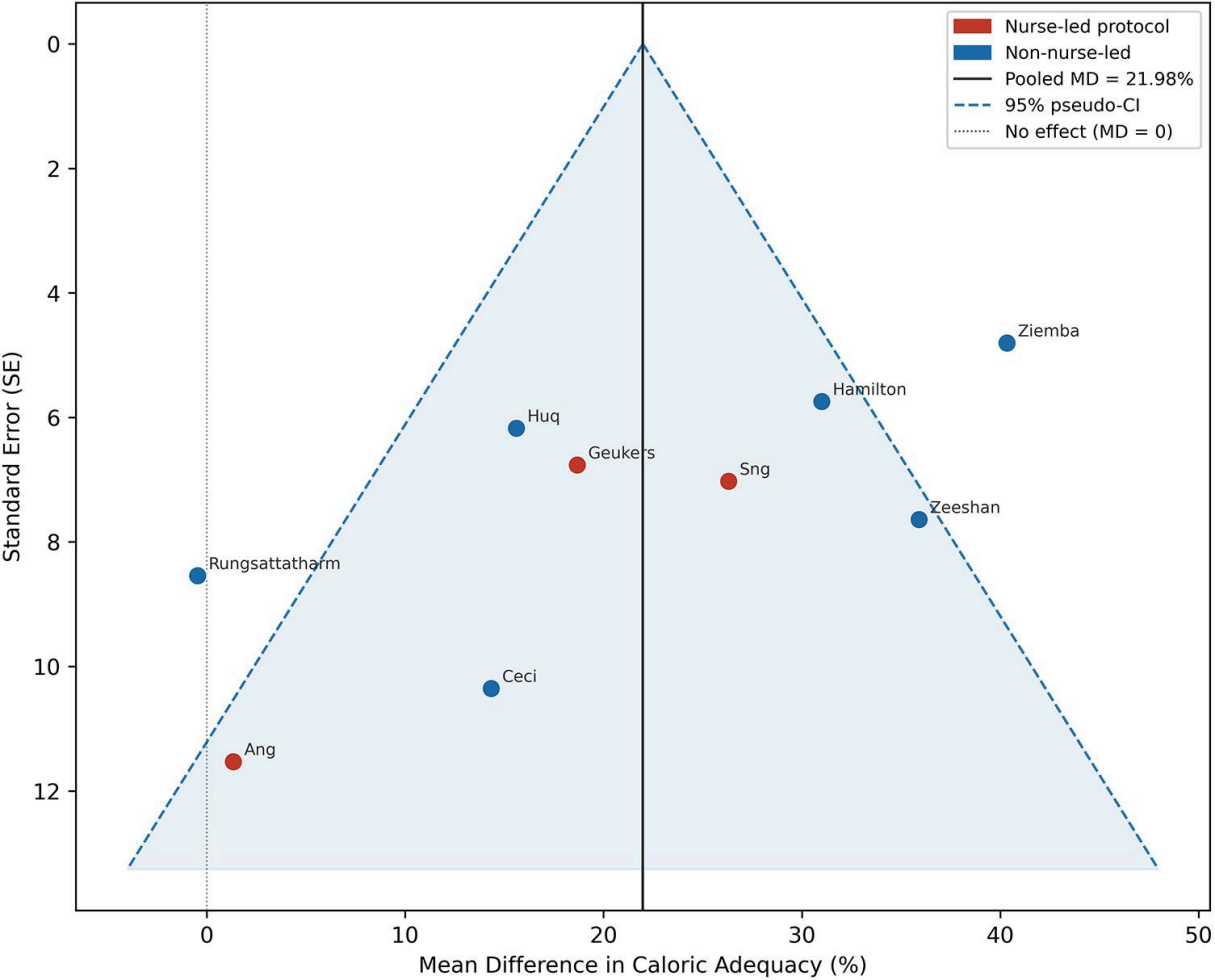
Funnel plot assessing small-study effects and publication bias. Funnel plot of included studies (*n* = 9) showing the relationship between effect size (mean difference in caloric adequacy, %) and study precision. The vertical solid line represents the pooled effect estimate (MD = 21.98%), while the dashed diagonal lines indicate the 95% pseudo–confidence limits. The dotted vertical line marks the line of no effect (MD = 0). Studies are visually differentiated by protocol type (nurse-led vs. non–nurse-led).

Visual assessment of the funnel plot (see Figure 4) suggested that there was a somewhat greater lack of smaller studies on the left of the funnel. A few smaller studies were more scatter to the negative side, and a few larger studies were at the right end, meaning that they reported more significant effect sizes than the other larger studies. This trend provides a general indicator of the possibility of small study effects, but not necessarily of publication bias.

Egger's regression test supported this observation and demonstrated statistically significant asymmetry (intercept = −5.40, SE = 1.93; *t* = −2.79; *p* = 0.0269). In practical, the asymmetry could just as easily reflect clinical heterogeneity, particularly given the earlier finding that study design strongly influenced effect size.

Furthermore, trim-and-fill analysis revealed no missing studies to impute (*k*₀ = 0), therefore, the pooled estimate remained unchanged (MD = 21.98%, 95% CI 13.38 to 30.59). Despite the statistically weak signal from Egger's test, there is no strong evidence that unpublished or missing studies are materially distorting the overall effect. Therefore, the observed asymmetry is driven by differences in study design and execution rather than selective publication alone.

### Sensitivity analysis

A series pre-specified sensitivity analyses (Table 3) were conducted to assess the robustness. Overall, the direction and statistical significance of the effect remained consistent across all analyses, though the magnitude varied in clinical contexts. For instance, leave-one-out meta-analysis showed that no single study materially altered the overall estimate and the pooled effect ranged from 19.40% to 24.99% after sequential omission, with all models remaining statistically significant (*p* < 0.001 throughout). When restricted by study design, a clear divergence emerged. Interventional studies (*k* = 4) demonstrated a substantially larger effect size (MD = 34.54%, 95% CI 28.66 to 40.42; I^2^ = 7.5%), with near-complete resolution of heterogeneity. In contrast, observational studies (*k* = 5) showed a more modest, though still significant, effect (MD = 12.14%, 95% CI 5.15 to 19.13; I^2^ = 8.0%). This nearly threefold difference in magnitude aligns with the meta-regression findings and suggests that study design is a key determinant of effect size.

**Table 3:** Key sensitivity analyses assessing robustness of pooled effect.

Sensitivity analysis	*k*	MD (%)	95% CI	*p*-value	*I*^2^ (%)
Primary analysis	9	21.98	13.38 to 30.59	<0.001	73.9
Leave-one-out	8	19.40–24.99	All significant	<0.001	60.9–77.2
Interventional only	4	34.54	28.66 to 40.42	<0.001	7.5
Observational only	5	12.14	5.15 to 19.13	0.0007	8
Standard care comparator only	5	24.09	15.68 to 32.49	<0.001	58.7
Fixed-effect model	9	25.25	20.75 to 29.75	<0.001	73.9

Taken together, these analyses confirm that the observed improvement in caloric adequacy is robust across multiple assumptions. The most significant source of variability seems to be with the study design and not the comparator definitions, clinically, this suggests that there is variability in how interventions are delivered and not whether they are effective.

## Discussion

Nutritional protocols significantly improved caloric adequacy in critically ill pediatric patients, yielding a mean difference of +21.98 percentage points (*p* < 0.0001) across 9 studies enrolling 1,992 children. This clinically meaningful signal underscores the potential of structured interventions to bridge the persistent gap in nutrient delivery. However, substantial heterogeneity was present (I^2^ = 73.9%). Further analysis showed that heterogeneity in design choice of protocols, patients and primary outcomes assessed likely affected this observed heterogeneity and would require a nuanced interpretation of the pooled effect. Specifically, the degree to which these protocols involved a multi-team approach, formed standardized sets of orders, or had standardized systems for automated caloric tracking may explain differential effectiveness.

According to Jouancastay et al. (3),, in control arms, caloric adequacy typically averages 52%–61% of targets, parallel to real-world PICU delivery reported as 45%–66% (3), such that the +22% absolute improvement elevates averages to approximately 74%–83%, approaching European Society of Paediatric and Neonatal Intensive Care (ESPNIC) (4) and American Society for Parenteral and Enteral Nutrition (ASPEN)/ Society of Critical Care Medicine (SCCM) (3) guideline targets of 60%–100%. That translates over an average of 7 days in the PICU to about 1.5 extra equivalent days at full caloric targets. Despite the observed heterogeneity, the consistent trend towards improved caloric intake reinforces the utility of structured approaches in optimizing nutrition support for this vulnerable population (2). Also, there is a direct relationship between achieving early and adequate amounts (at least 60% of energy targets during the first 7 days) of delivered calories and subsequent 60-day mortality reduction in mechanically ventilated children, and no observed additional infections or decreased ventilator-free days (2).

We also clarify the effects of the planned nutritional interventions, namely working out the mechanisms according to which protocolized feeding reduces the physiological response to stress and accelerates the activity of organs in children in the ICU (28). The focus on initiation of early enteral nutrition is very essential as the effect of underfeeding is deleterious to greater morbidity and mortality (29). In particular, feeding protocols that use volumes have been shown to be more effective in energy delivery and there are studies that show that energy delivery improvement in intervention groups is better in volume-based feeding protocols (30). This method systematically responds to a caloric deficit, distinguishing it over the classical types of weight-based feeding practices (30). This result is consistent with the past studies that have indicated the significance of structured programs in encouraging regular and optimal nutrition provision (31).

We depicted point estimates that marginally supported the non-nurse-led protocols in our research but the test between the groups was insignificant (*p* = 0.51). This impounds that though the role of nurse intervention is fruitful, other systematic methods can be as effective as nurse intervention in maximizing the caloric input. This subtle observation means that it is the systematized, logical use of nutritional measures, and not the professional occupation they are implemented with, that is the main determinant of success. The results are consistent with the reported effectiveness of high-energy nutrition regimens to lessen weight decline in the critically ill infants, as was reported in a study of 59 post-cardiac surgery infants in which the intervention group feeding on high-energy nutritional regimen experienced a markedly lesser weight loss in comparison to controls on standard formula feeding (32). This is also justified by the volume-based feeding protocols, which improve the efficacy of energy delivery with more significant improvements in the intervention groups (MD = 15.41%, *p* < 0.001) (30), and early enteral nutrition, which narrows the hospital stay (33).

These organized practices therefore enhance development and curb the tendencies of underfeeding such a risky group (29). However, uniform feeding guidelines, irrespective of the lead professional, are always associated with enhanced time to full enteral volume which is paramount to facilitate growth and averting undesirable consequences on the susceptible pediatric groups (34). The topic of early enteral nutrition is especially pertinent, since research studies indicate that EEN could decrease in-hospital mortality and hospital stay among critically ill children, yet the validity of this finding remains insufficient (35).

It should be mentioned that the lower levels of heterogeneity in the nurse-led approaches were significantly lower (I^2^ = 41.7% vs. 79.8%). This implies higher implementation consistency that would mean consistent implementation of nutritional guidelines on the implementation side once empowered nurses take the lead in the feeding process (34). The incorporation of computerized provider order entry systems into these protocols that are led by nurses has also shown an improved ability to maximize nutrient delivery (36). This consistency makes nurse empowerment a powerful clinical and systems level tool to optimize nutrition and was supported by PICU research showing an increase in energy and macronutrient delivery through nurse-based feeding algorithms and protocols. The resulting decrease in heterogeneity also justifies the formulation of standardized nurse-led feeding guidelines as an essential initiative to improve the consistent and effective provision of nutritional care in the PICU (37).

Subgroup analysis on the basis of standard care (MD +24.09) vs. historical control (MD +19.60) comparators showed absence of significant between group difference (*p* = 0.61). The uniformity of the different types of protocols regardless of the specific one, or the dominating professional group, indicates a major benefit of the systematic nutritional management vs. the unstructured approach in the PICU setting (38). The design of historical control is not dependent on the intervention and prone to inflation of secular trends wherein, as nutrition care improves over time, the benefits of protocols are overestimated. As such modern randomized controlled trials are necessary to accurately determine the real effectiveness of new nutritional protocols. Moreover, the assessment of caloric adequacy should be made based on the quantity and quality of nutrients, as well as timing since the results of studies on the relationship between early achievement of nutritional goals and the better clinical outcome include lower infection rates and shorter ventilator days (5). But our sensitivity analysis which is limited to the standard care comparators (*k* = 5, MD +24.09%, *p* < 0.0001, I^2^ = 58.7%) confirms that the positive effect still exists in a more rigorous set of conditions which effectively excludes the secular trends as the main factor. This robust evidence base highlights the critical role of well-implemented protocols in enhancing caloric adequacy in critically ill children.

Meta-regression analyses indicated that age (*β* = +0.07/month, *p* = 0.68), enteral feed route (*β* = +4.47%, *p* = 0.71), study duration (*β* = +1.56%/year, *p* = 0.57), and sample size (*p* = 0.11) did not significantly moderate the intervention effect on caloric adequacy. However, a positive, albeit non-significant, association between caloric adequacy and the proportion of enteral feeding highlights the potential for enhanced nutritional delivery via the gastrointestinal route. This observation aligns with prevailing guidelines advocating for early and progressive enteral nutrition as the preferred method for nutrient delivery in critically ill children, even in the presence of vasoactive support, due to its multifaceted benefits beyond caloric provision, including gut integrity maintenance and immunomodulation (39). The optimal protein intake, considering energy provision, also remains an area of active investigation, as current evidence suggests potential benefits of higher protein delivery for clinical and nutritional outcomes in this vulnerable population (40). Such non-significant results are promising, because they support the strength and inter-group reliability of protocol benefits in neonates as well as older children, enteral routes of nutrition (nasogastric and gastric), and the duration of the studies and protocols, highlighting the high effectiveness and sustainability of structured nutritional programs in various PICU settings (4, 41). Additional studies are required in order to outline the exact mechanisms by which the enteral nutrition has an effect on clinical outcomes, more specifically the meshing-up of the macro and micronutrient inoculation and the gut microbiome adjustment among the critically ill children patients (6). Regardless of these improvements, there are still existing issues, such as high levels of malnutrition and deficiencies in meeting of caloric goals, especially in patients undergoing extracorporeal membrane oxygenation or high-dose vasoactive care (42). The research that ought to be done into the future must consequently be aimed at creating and verifying new and efficient nutritional programs and feeding regimes that can help address these physiological inhibitions and reliably maintain caloric sufficiency in the sickest pediatric patients.

Our meta-regression (*k* = 9) in our study was underpowered, and all covariate analyses are exploratory. The major heterogeneity in meaning of caloric adequacy and construction of protocols used in four studies, in addition to the prevalence of observational designs, dependence on historical controls, makes the pooled estimate less precise. Importantly, patient centred outcomes e.g., ventilator free days, infection rates, and mortality, ended up being inconsistently reported to allow secondary pooling. Results cannot be safely extrapolated outside of well-staffed, committed PICUs.

Multicentric controlled randomized trials with powering of clinical endpoints should be an adequate objective in future studies as process outcomes alone are not sufficient. Standardized concepts of caloric adequacy, future fidelity measurements, and extended follow-up studying lean mass recoveries and neurodevelopment patterns are required. The benefit of reproducibility observed among nurse-led protocols deserves specific implementation science investigation. Our study represents the first meta-analysis specifically evaluating structured nutritional protocols in PICU patients. However, certain limitations remain. The number of included studies was small, substantial heterogeneity was present, definitions of caloric adequacy varied across studies, and most studies focused on process outcomes rather than patient-centered clinical outcomes. Illness severity scores (e.g., PRISM, PIM, pSOFA) were not uniformly reported across included studies and could not be incorporated into meta-regression analyses; future trials should prospectively collect and report standardized severity indices to enable severity-adjusted effect estimates.

## Conclusion

In nine studies, structured nutrition interventions achieved an average 22 percentage-point increase in caloric adequacy, a difference between persistent underfeeding and achieving guideline-recommended nutrition goals. Interestingly, the benefit was not dependent upon the provider of the protocol; nurse-led and clinician-led methods worked equally well. There was enough heterogeneity to be considered substantial but there was a clear and understood explanation for this heterogeneity with the majority of the between study variance attributable to differences in study design and the direction and significance of the effect was maintained in all sensitivity analyses. The results of this study strongly support the recommendation of using formalized feeding regimens as a standard of practice for PICU patients. They put nurse empowerment at the heart of a practical and scalable solution to provide more uniform delivery of nutrition to one of the most vulnerable patient groups in hospitals.

## Data Availability

The original contributions presented in the study are included in the article/Supplementary Material, further inquiries can be directed to the corresponding author/s.
